# Trends and Advances in the Characterization of Gas Sensing Materials Based on Semiconducting Oxides

**DOI:** 10.3390/s18103544

**Published:** 2018-10-19

**Authors:** David Degler

**Affiliations:** European Synchrotron Radiation Facility, 71, Avenue des Martyrs, 38043 Grenoble, France; david.degler@esrf.fr; Tel.: +33-476-88-1925

**Keywords:** gas sensor, material characterization, in-situ/operando, complementary techniques, active/inactive species

## Abstract

The understanding of the fundamental properties and processes of chemoresistive gas sensors based on semiconducting metal oxides is driven by the available characterization techniques and sophisticated approaches used to identify structure-function-relationships. This article summarizes trends and advances in the characterization of gas sensing materials based on semiconducting metal oxides, giving a unique overview of the state of the art methodology used in this field. The focus is set on spectroscopic techniques, but the presented concepts apply to other characterization methods, such as electronic, imaging or diffraction-based techniques. The presented concepts are relevant for academic research as well as for improving R&D approaches in industry.

## 1. Introduction

The development of chemoresistive gas sensors based on semiconducting metal oxides (SMOX) is driven by innovations in material science [1,2,3,4,5,6], miniaturization of sensor devices [7,8] and understanding of the gas sensing process itself [9,10,11]. The advances in miniaturization strongly decreased size, production costs and power consumption of SMOX gas sensors making SMOX-based sensors attractive for mobile applications and technologies related to the Internet of Things [7,12,13]. Despite the large number of publications reporting the gas sensing properties of various SMOX-based materials, the lack of selective gas sensing materials remains as a major challenge for many gas sensor applications.

The development of gas sensors is still based on trial and error rather than on knowledge-based approaches, which enable a rational design of sensing materials based on the understanding of fundamental processes [14]. The fundamental understanding involves a detailed knowledge on the relationship of structures, properties and functionality (structure-function-relationship) as well as on the connection of synthesis routes and obtained structures. Thus, it is essential to determine correctly structures and properties, and to associate these with gas sensing performance or synthesis routes. This approach requires suitable materials characterization techniques. The large variety of spectroscopic techniques allows assessing various structural, chemical and electrical properties. Spectroscopic techniques therefore form a major part of the tool-box used to study SMOX-based gas sensing materials [15,16]. To determine correctly structure-function-relationships attention should be drawn to the most suitable approaches for material characterization by spectroscopic and other techniques. Important aspects of the characterization of SMOX gas sensing materials and the investigation of gas sensing mechanisms are summarized in the following sections with a focus on experimental methodology, use of multiple and complementary techniques and appropriate identification of species actively involved in the gas sensing process.

## 2. In-Situ and Operando Methodology

An essential aspect of studying SMOX gas sensing materials is the question whether one can relate the gained information to what is accentually happening during gas sensing in real conditions. For decades, the fundamental processes of SMOX-based gas sensors were studied by surface science techniques applied to idealized samples, such as single crystal surfaces [17,18]. The used surface science techniques typically involve incoming and/or outgoing electrons and, thus, ultrahigh vacuum conditions. Furthermore, most of these works were conducted at room temperature or even below [19,20]. Although many of these methods allow the study of well-defined systems, the obtained results are far from realistic conditions. This results in three major gaps between the experimental and realistic operation conditions, namely in material, pressure and temperature [21].

The material gap arises from the fact, that single crystal surfaces may not adequately represent actual sensing materials, which are typically pristine, doped or loaded nanoparticles forming a porous layer. The properties of the nanoparticles as such may widely differ from the ones of a certain single crystal surface. Furthermore, morphological properties of SMOX layers, such as grain-grain-boundaries, are essential for the gas sensing process [9,22].

The pressure gap is described by two aspects. The most obvious one is the several orders of magnitude difference between atmospheric pressure (1013 mbar) and high vacuum (10^−7^ mbar or less), which has a major impact on adsorption and surface chemistry and, thus, especially on the gas reception process. Related to this aspect is the partial pressure, i.e., the concentration, of the reactive gases, which should be present in a realistic concentration. In ambient applications, gas sensors are used to detect traces of analytes in the ppm and ppb range in the presence of around 20 vol% oxygen and 20–80 %r.h. (absolute 0.5 to 2.5 vol% water vapor at 25 °C) [16,22]. These concentrations should be considered when studying SMOX gas sensors, i.e., when measuring the gas sensing performance or conducting further research, e.g., by spectroscopic techniques.

The temperature gap has a strong impact on the semiconducting properties and the surface chemistry of the gas sensing material, as the ionization of defects [23,24], adsorption of molecules [25,26] or chemical reactions [27,28] strongly depend on temperature. Heating the sample to typical operation temperatures between 200 and 400 °C is possibly restricted by the spectroscopic technique as such, e.g., requiring ultralow temperatures or by low pressures, which my trigger unwanted changes in the sample due to heating in the absence of oxygen.

Studying gas sensing materials under realistic operation conditions is essential for understanding their working principle but extending the experimental conditions beyond the actual application conditions may provide additional insights, e.g., providing trends with temperature or gas concentrations, or allow a better understanding of certain material aspects by studying a specimen which is less complex than a porous layer of nanoparticles. Nevertheless, for establishing structure-function-relationships is mandatory to obtain the anticipated information as close to real operation conditions as possible, to determine correctly obtained structures or identify active centers. For various functional materials, using so-called in-situ or operando methods is considered as state of the art approach for gaining insights in fundamental working principles [15,16,21,29]. In the early 2000s, various researchers in the field of catalysis coined the term operando. The details of the definitions are still a matter of debate and vary for different functional materials. In general, the following definitions can be considered [15,16]:

- Ex-situ studies may include all types of specimens and experimental conditions. However, in many cases gaps in material, pressure and temperature are encountered. An example is the study of sensing material as powders at room temperature by using typical vacuum techniques.
- In-situ studies are performed in more appropriate conditions, namely temperatures, pressures and atmospheric compositions which match real operation conditions. These measurements might be carried out on various types of specimens.
- Operando studies are performed under application relevant conditions and involve the real-time evaluation of the performance of the studied material. In case of a gas sensor the performance evaluation is the sensor response/signal, while for a catalyst the performance in evaluated by catalytic conversion. The real-time evaluation of the performance implies that the study is done on a real device, e.g., a gas sensor. Thus, operando studies close the three gaps between laboratory experiment and application as much as possible.

**Figure 1:**
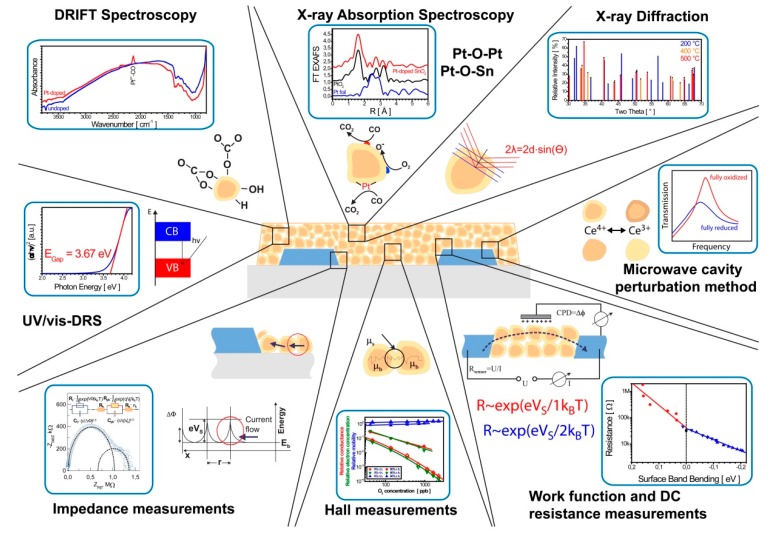
Selection of available in-situ and operando methods for studying SMOX gas sensors including different techniques ranging from spectroscopies over diffraction to electrical measurements. The following techniques are shown (clockwise): XRD [31], microwave perturbation measurements [43], simultaneous work function and DC resistance measurements [44,45,46], Hall effect measurements [47,48,49], impedance measurements [22,39], UV/vis-DRS [35,36], DRIFTS [28,39,40,41], XAS/XES [34,37,38]. The figure is reprinted from reference [14].

Studying gas sensors during operation by means spectroscopic methods emerged in late 1990s and developed in the following decades [15,30]. As numerous as the processes related to gas sensing with SMOX are, so versatile are the methods that are used to study gas sensing materials. Structural properties are studied by X-ray diffraction (XRD) [31] or X-ray absorption (XAS) [32,33,34]. The electronic structures can be probed by UV/vis Diffuse Reflectance Spectroscopy (DRS) [35,36], XAS and X-ray emission spectroscopies (XES) [34,37,38]. The surface chemistry is mainly investigated by Diffuse Reflectance Infrared Fourier-Transform Spectroscopy (DRIFTS) [28,39,40,41] and Raman spectroscopy [27,42]. An overview of operando methods for SMOX gas sensors is given in Figure 1 [14]. A detailed discussion of in-situ and operando spectroscopic techniques is given elsewhere [15,30].

Operando spectroscopies are powerful tools for studying functional materials, such as gas sensing materials, but there are limitations. If the changes caused by the interaction with a target gas are rather small, the observable effects are possibly close to the noise level and the required signal to noise ratio is not necessarily reached during an operando experiment. In many cases operando spectroscopies provide a detailed picture of gas sensor during operation, however one major challenge is to identify structures and spices that are actually related to the gas sensing process (see Section 4). Furthermore, the experiment itself can influence the materials or gas sensing process when using radiation, which excites, changes or damages the material, e.g., electronic transitions excited by UV and visible light or reduction of the sample by hard X-rays.

**Figure 2:**
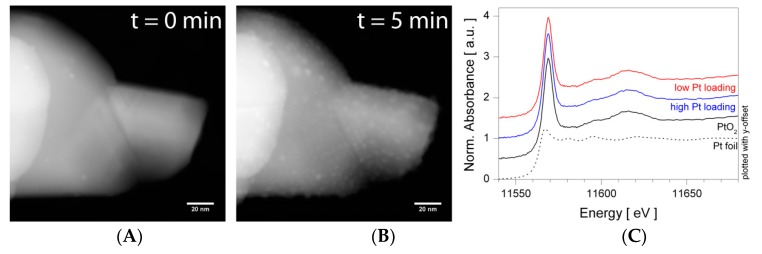
Material characterization of highly Pt loaded SnO_2_ by high-angle annular dark-field STEM (**A**,**B**) and X-ray absorption near edge structure (XANES) spectra of the differently concentrated Pt loaded SnO_2_ samples and reference compounds (**C**). Both methods were applied to ex-situ samples at room temperature, but at different pressure, i.e., vacuum for STEM and ambient air for XAS. Figures are reprinted from reference [32], reproduced by permission of The Royal Society of Chemistry.

The operando methodology is commonly used to investigate structure-function-relationships, but it is important to consider the pressure and temperature gaps when characterizing materials for instance by means of transmission or scanning electron microscopies (TEM/SEM) or by photoelectron spectroscopies. The pressure gap of several orders of magnitude can result in unwanted structural changes directly or induced by the measurement itself. In case of Pt loaded SnO_2_, scanning transmission electron microscopy (STEM) shows the presence of metallic Pt clusters on the SnO_2_ surface (Figure 2A,B), structural investigations by XAS indicated the presence highly oxidized Pt (Figure 2C). Repeatedly measuring the same area by STEM showed a steady increase of the metallic Pt clusters, indicating that they are formed during the measurement [32]. In addition to that, operando XAS shows only the presence of oxidized Pt during sensor operation at 300 °C in synthetic air and at normal pressure. The oxidized Pt loadings on SnO_2_, however, are easily reduced in the absence of atmospheric oxygen [50]. Apparently, both methods yield quite different results and having STEM results only, misleading conclusions about Pt structure would be obtained, since Pt is reduced due to the vacuum and the electron beam. Both methods combined support the conclusion that oxidized Pt forms surface clusters, which are easily reduced to metallic Pt. This example emphasizes two important aspects for material characterization:

- The material characterization should be done as close as possible to real operation conditions, by minimizing gaps in pressure and temperature. Even if the characterization is still considered to be ex-situ, like in the shown example (Figure 2), minimizing these gaps provides more accurate information.
- Using several techniques to assess similar aspects provides further advantages: Independent results allow verifying the drawn conclusion, e.g., on the structure, and in many cases complementary information is obtained, e.g., STEM offers spatial information on the distribution, while XAS is feasible operando technique, which allows assessing the structure under various conditions.

## 3. Complementary Techniques and Multi-Probe-Approach

A holistic picture of a gas sensing material is obtained by using multiple and complementary techniques. The use of complementary techniques in a multi-probe-approach is commonly used for the investigations of various catalysts. The information might be recorded in separate experiments or at the same time [21,51,52,53]. For spectroscopic and other characterization techniques, the term ‘complementary’ includes techniques, which probe different properties, a similar kind of property or properties at different length or depth scales. Each of these aspects be relevant.

There are various examples for the study of different properties, e.g., the measurement of surface species by DRIFTS, residual gas analysis of reaction products by photo-acoustic IR spectroscopy and work function changes by a Kelvin probe setup during C_3_H_6_ sensing in two different experiments [54]. Such a multi-probe-approach is of fundamental importance when investigating the complex gas detection mechanism of a material, which involves chemical and electrical processes, which cannot be detected by a single method.

**Figure 3:**
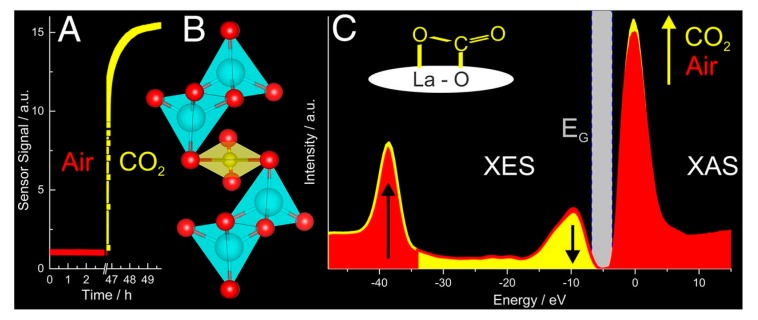
Operando spectroscopic study of La_2_O_3_CO_3_ during CO_2_ (10,000 ppm) sensing in 50 %r.h. at 250 °C: (**A**) Sensor signals, (**B**) schematics of the unit cell of La_2_O_2_CO_3_ (La = cyan, O = red, C = yellow) and (**C**) XES and XAS spectra before (red) and during (yellow) CO_2_ exposure. Figure is reprinted from reference [38].

Another connotation of complementary is used for different spectroscopic techniques probing similar properties but providing only a partial picture. IR and Raman spectroscopies are typical textbook example; the different selection rules result in the measurement of different molecular vibrations and thus different species [55]. When probing the electronic structure by X-ray spectroscopies, the unoccupied states are probed by XAS, while the occupied states are probed by XES [56,57], i.e., XAS and XES provide complementary information on the electronic properties and combining results in a complete picture of the electronic structure, e.g., as shown for La_2_O_2_CO_3_-based gas sensing materials [38]. The complementary information of the X-ray emission and absorption spectra (Figure 3C) show the oxidative character of CO_2_ ionosorption on La_2_O_2_CO_3_ by the decrease of the emission from the highest occupied sates in the X-ray emission spectra (−10 eV) and by an increase of the transition into unoccupied states, which is indicated by the whiteline in the XANES spectrum (0 eV).

Many material characterization techniques are complementary in terms of the length or depth scale of the obtained information. This is of importance since gas sensing takes places on different length scales, ranging from chemical processes on the atomic level over space charge layers in the nanometer range to charge transport and diffusion processes on the micrometers scale [9,22]. Gas sensing materials present structures of different sizes, such as SMOX based materials forming clusters typically between 10 and 100 nm [58] and additives forming clusters of a few nm or less [32,37] and even single atom/ion sites [33,34]. Raman spectroscopy or XRD require large, crystalline and long-range ordered structures, while XAS is already sensitive to short-range ordered and amorphous structures and, thus, can be used to probe small clusters or incorporated atoms/ions, e.g., PdO clusters on SnO_2_ or Pd ions incorporated in SnO_2_ [34,59]. Complementing methods which probe long-ranged ordered samples typically the SMOX based material and unordered structures in many cases additives provides a full picture of the material’s structure.

In addition to size and length scales, the surface and bulk properties and differences between those properties play an important role for gas sensing. The gas reception by SMOX is mainly related to surface processes [27,60,61], but bulk properties of the SMOX have a huge impact on the electronic properties and charge transport within the material which has a strong impact on the transduction mechanism [9]. Despite the importance of properly characterizing surface and bulk properties, this aspect is commonly not considered. Using complementary techniques providing mainly surface or bulk information, would provide a more detailed picture and better understanding of gas sensing properties.

**Figure 4:**
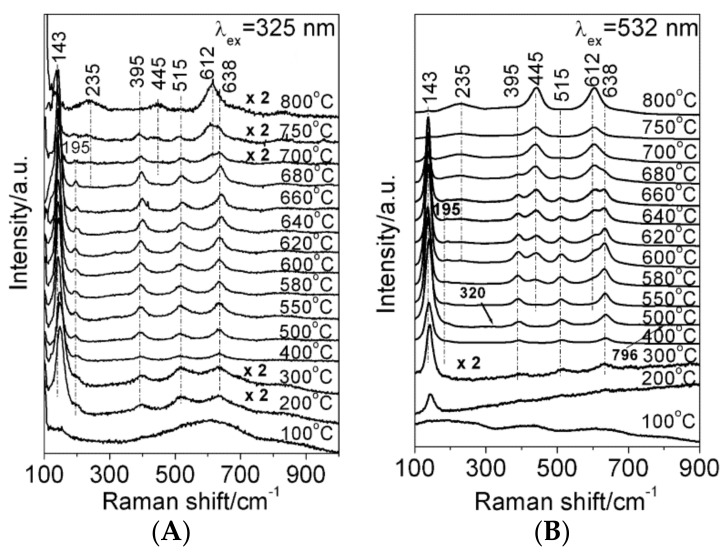
Raman spectra of TiO_2_ calcined at different temperatures. (**A**) The spectra were recorded with an excitation at 325 nm, (**B**) with 532 nm. Reprinted with permission from The Journal of Physical Chemistry B, reference [63]. Copyright 2006 American Chemical Society.

There are several reports that demonstrated to feasibility of probing surface and bulk properties of the same material: A successful operando spectroscopic approach is the combination of bulk sensitive Raman and surface sensitive DRIFT spectroscopy for the study of Pt-Ba/CeO_2_ during NO_x_ storage reduction [62]. Another interesting approach in the field of SMOX is recording Raman spectra with different incident wavelengths, at energies below or above the optical band gap providing bulk or surface information, respectively [63]. This approach can be used to determine differences in between surface and bulk of nanoparticles, e.g., transitions of the crystal structure. Figure 4 shows a series of Raman spectra of TiO_2_ calcined at different temperatures, which were recorded with an incident wavelength of 325 and 532 nm, respectively. The Raman bands at 395 and 445 cm^−1^ correspond to the anatase or rutile phase, respectively. With the bulk sensitive excitation wavelength (532 nm) one observes the transition from anatase to rutile between 580 and 680 °C, while with the surface sensitive excitation wavelength (325 nm) one observes this transition at 750 °C.

## 4. Identifying Active and Inactive Species

Establishing structure-function-relationships for SMOX gas sensors requires the identification of active sites and active species. During an operando experiment, one detects various surface species and changes of the material, but not all detected species are actively involved in the gas sensing process. For example, the decrease of surface hydroxyl groups on SnO_2_ during the exposure of reducing gases (CO, H_2_), which is not directly caused by an interaction of the reducing gas with the hydroxyl groups but rather related to a subsequent rearrangement between the equilibrium of surface oxygen and hydroxyl groups [41,60]. Another example is the formation of carbonate species on SnO_2_ during CO sensing, which are assumed to be intermediates of the gas reception mechanism, e.g., formed by the reaction with molecular oxygen (Reaction (1) and Reaction (2)) [64]:

(1) $$\mathrm{CO}+{\left(O_{2}\right)}_{\mathrm{ads}}^{\alpha -}\to {\left({\mathrm{CO}}_{3}\right)}_{\mathrm{ads}}^{\alpha -}$$

(2) $$\mathrm{CO}+{\left({\mathrm{CO}}_{3}\right)}_{\mathrm{ads}}^{\alpha -}\to 2\cdot {\mathrm{CO}}_{2}+\alpha \cdot e^{-}$$

Recent theoretical and experimental works show that the CO reception on SnO_2_ takes place by a reaction of CO with surface lattice oxygen, which creates an oxygen vacancy and releases electrons into the conduction band (Reaction (3)) [60,65,66]. The formation of carbonates was found be related by the subsequent reaction of CO_2_, which is formed by the oxidation of CO, with surface oxygen (Reaction (4)) [60]. While the reaction of CO with surface oxygen releases electrons to the conduction band, i.e., including changes in the surface charge, the reaction of CO_2_ does not change the surface charge and, thus, the latter reaction is an inactive spectator species.

(3) $$\mathrm{CO}+O_{O}^{}\to {\mathrm{CO}}_{2}+V_{O}^{\alpha +}+\alpha \cdot e^{-}$$

(4) $${\mathrm{CO}}_{2}+O_{O}^{}⇋{\left({\mathrm{CO}}_{3}\right)}_{O}^{}$$

The challenge of identifying active species is well-known from catalysis research, since not all detected surface species contribute to the catalytic process and often correspond to slowly reacting or completely inactive spectators [21,67,68,69]. In the case of gas sensing, similar considerations can be made:

- Active species and processes change the concentration of surface charge as a result of changes in the atmosphere;
- Inactive species do not change the surface charge or are not involved in processes changing the surface charge.

**Figure 5:**
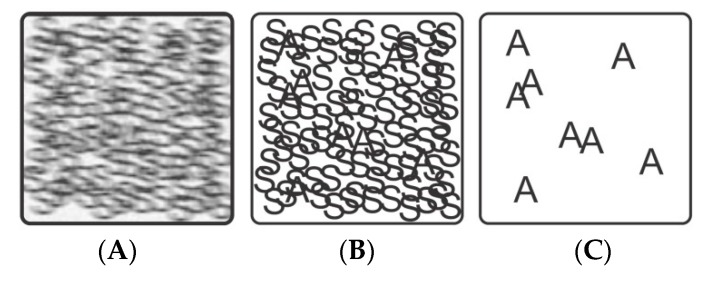
Three situations found by in-situ and operando spectroscopy: (**A**) realistic, (**B**) sensitive and (**C**) sensitive and selective. ‘A’ represents active species; ‘S’ represents inactive spectator species. The figure is reprinted from reference [70].

The reaction rate as such does not necessarily indicate whether a species is active or inactive for gas sensing, but for an active species the reaction rate will strongly influence response and recovery time. Properly conducted in-situ and operando experiments will provide a picture of all present species or changes in properties during gas sensing. As indicated in Figure 5A, in a realistic situation many active and inactive species are found and a clear assignment is limited by different factors, such as noise or low resolution [70]. Using more sensitive methods will help to improve obtained data (Figure 5B) and using selective techniques or sophisticated approaches one increases the selectivity of assessing active species (Figure 5C).

There are various approaches to increase the sensitivity and selectivity of experimental techniques. Most of the methods involve time-resolved measurements, which allow correlating of the observed species with the performance of the material, e.g., formed products (catalysis) or electrical response (gas sensing). However, already selecting a suitable experimental technique or improved approach offers various possibilities. If the site of interest is related to one specific element, e.g., a noble metal additive in a gas sensing material, element selective techniques such as XPS, XAS or XES can provide site selective information [38,71]. In case of vibrational spectroscopies, using isotopically labelled analyte gases (H/D, ^12^C/^13^C, ^14^N/^15^N, ^16^O/^18^O) allows an improved identification of surface species related to the analyte [41,60]. Still, using the most suitable technique as does not ensure a proper identification of active species. The identification is strongly improved, if one is able to compare the evolution of surface species with the sensor signal, i.e., in case measurements are done with sufficient time resolution. Using a combination of time resolved DRIFTS and impedance measurements, conductivity changes in ZSM-5-based catalysts during the selective catalytic reduction of nitrogen oxides were successfully assigned to a certain adsorbed NH_3_ species, by comparing the time constants of the electrical response and the different adsorbed NH_3_ species [39]. Other approaches using time-resolved methods are Modulation Excitation Spectroscopy (MES) [70,72,73,74], Steady State Isotopic Transient Kinetic Analysis (SSITKA) [68,75] and the evaluation of time-resolved data by Multivariate Curve Resolution (MCR) [76,77]. These rather sophisticated approaches are applied by various authors to study catalysts, but today there is just a small number of articles dedicated to the investigation of gas sensing materials [28,39,73,78]. A good example is the investigation of the NO_2_ detection of In_2_O_3_, using MCR to analyze time resolved DRIFT spectra [28].

**Figure 6:**
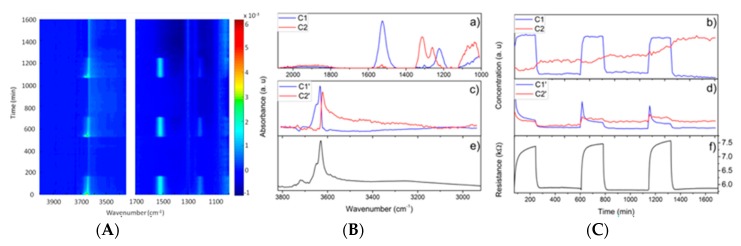
DRIFTS investigation of the NO_2_ detection mechanism of In_2_O_3_: 2D-representation of time resolved DRIFT spectra (**A**) of an In_2_O_3_ gas sensor alternatingly exposed to 1 ppm NO_2_ (3 h) and air (6 h) at 350 °C. Component spectra (**B**) and concentration profiles and sensor response (**C**) derived from the time-resolved operando DRIFTS experiment. A further explanation of the component spectra and concentration profiles is given in the original publication. Reprinted with permission from ACS Sensors, reference [28]. Copyright 2017 American Chemical Society.

The DRIFT spectra recorded during NO_2_ sensing at 300 °C with pristine In_2_O_3_ (Figure 6, left) show a series of bands, which respond to the presence of NO_2_ (1221 and 1520 cm^−1^), and bands, which remain at the surface after the first NO_2_ pulse and increase with each NO_2_ pulse (1313 and 1260 cm^−1^). Already the visual inspection of the spectra suggests the presence of active species, i.e., species causing a resistance change, and spectator species. The bands at 1221 cm^−1^ and the ones at 1313 and 1260 cm^−1^ correspond to nitrite and nitrate species, respectively. The band at 1520 cm^−1^ can be assigned to both nitrites and nitrates. The component spectra (Figure 6, center) and concentration profiles (Figure 6, right) obtained by MCR show a clear difference for the nitrite and nitrate species: The nitrite species correlate with the electrical response, while the nitrates accumulate over time. Based on these findings it is concluded, that the ionosorption of NO_2_ on In_2_O_3_ (Reaction (5)) changes the surface charge, i.e., is actively involved in gas sensing, while the formation of nitrates is related to a subsequent reaction of ionosorbed NO_2_ with surface lattice oxygen (Reaction (6)):

(5) $${\mathrm{NO}}_{2}+S+e^{-}⇋{\left({\mathrm{NO}}_{2}\right)}_{S}^{-}$$

(6) $${\left({\mathrm{NO}}_{2}\right)}_{S}^{-}+O_{O}⇋{\left({\mathrm{NO}}_{3}\right)}_{S}^{-}$$

The example of the NO_2_ detection mechanism on pristine In_2_O_3_ shows, that an appropriate assignment of active species and spectator species is possible based on time resolved spectroscopy and chemometric data analysis. With increasing complexity of sensing materials, e.g., doped and loaded SMOX, a more complex chemistry of analyte gases, e.g., oxidation of volatile organic compounds, and more complex atmospheric compositions, the identification of active species will be more and more difficult, but essential for determining structure-function-relationships. Thus, advanced methods and well-planned experiments will play an increasingly important role for the understanding of SMOX-based gas sensing materials.

## 5. Conclusions

Understanding the relationship of structures, properties and gas sensing performance is essential for the knowledge-based design of new, tailor-made gas sensing materials. The methods available for the investigation of SMOX-based gas sensors reached an unprecedented variety, which allows to probe various structural, electrical and chemical properties of SMOX gas sensing materials in realistic conditions and in real-time. Simply reporting the sensing response and ex-situ material characterization is an insufficient basis for the understanding of the gas sensing process. The advanced material characterization techniques allow a much more reliable and detailed study of SMOX-based gas sensing materials. The key aspects presented in this article can be summarized as follows:

- A proper material characterization should be done as close as possible to the real operation conditions, i.e., preferentially during sensor operation, and involve several complementary techniques.
- Mechanistic studies of the gas sensing process should be based on in-situ and operando methods, minimizing the effect of the material, pressure and temperature gaps. Special attention should be drawn to atmospheric compositions, which should match the ones in real application, especially in case of gas concentrations and interfering compounds, such as water vapor.
- Using complementary techniques in a multi-probe-approach allows to probe various properties, processes or species within the same experiment or a series of similar experiments. This involves techniques being complementary in information depth or in the nature of the probed properties.
- Determining structure-function-relationships requires to differentiate between active and inactive species. A proper design of the experiment, using selective techniques or time-resolved methods strongly enhances the ability to correctly identify active species.

The increasing knowledge on structure-function-relationships of SMOX-based gas sensing materials is ultimately driven by the continuously improving experimental methods available, which provide a more and more detailed picture of the various aspects of the gas sensing process. Further improving the available methods and systematically using state of the art approaches will allow to extent the understanding of SMOX-based gas sensors from a limited number of well-established model materials to a general understanding of fundamental properties and processes of SMOX gas sensing materials.
